# Miscellany

**Published:** 1906-09

**Authors:** 


### MISCELLANY.

The State Civil Service Commission announces that it will receive applications until September 4, 1906, for the position of Woman Inspector of Nurse Training Schools in the State Department of Education. The salary is from $1800 to $2100. Candidates must be citizens of the United States, legal residents of New York State, registered nurses and graduates of a registered nurse training school with at least five years experience since graduation in supervision, administration or instruction in a nurse training school.

Application forms and particulars of the competition may be obtained by addressing the Commissioner at Albany, of which Mr. Charles S. Fowler is the Chief Examiner.

---

In view of the meeting of the Military Surgeons which is to take place at Buffalo, September 11-14, 1906 it becomes of the first importance to see that the guests are well housed or quartered while visiting the city. The Iroquois is the leading hotel in the center of the business section and is unsurpassed by any hotel west of New York City either in rooms or cuisine, or service. In the residential sections The Touraine and The Lenox are hotels of the first order and are administered by men of experience who take high rank as courteous hosts.

In the business and railroad districts The Broezel and The Stafford are well and favorably known as excellent hotels of their class, the rates charged in these latter two being less than in the others named. In any event no mistake will be made by any tourist or visitor to the city who secures accomodations at either of the houses named.

